# Extreme Elevation of Creatine Kinase in a Young Male Patient With Recurrent Rhabdomyolysis

**DOI:** 10.7759/cureus.24817

**Published:** 2022-05-07

**Authors:** Layla Al Bizri, Anh Do, Daniel R Ouellette

**Affiliations:** 1 Pulmonary and Critical Care Medicine, University Hospitals Cleveland Medical Center, Cleveland, USA; 2 Department of Heart Failure and Transplantation, University of Alabama, Birmingham, USA; 3 Pulmonology and Critical Care, Henry Ford Health System, Detroit, USA

**Keywords:** medical intensive care unit (micu), creatinine kinase. acute kidney injury, acute renal failure and hemodialysis in icu, critical care and hospital medicine, non-traumatic rhabdomyolysis

## Abstract

Rhabdomyolysis is a common cause of admission to the intensive care unit. However, recurrent rhabdomyolysis remains a rare encounter for intensivists and presents a challenge in terms of identifying its etiology. Considerations of metabolic myopathies as a culprit remain underexplored. We present a case of a patient with recurrent rhabdomyolysis with extreme elevation of creatine kinase.

## Introduction

Rhabdomyolysis signifies injury to striated muscle cells, and its diagnosis can be made based on clinical symptoms and elevations in creatinine phosphokinase (CPK) [1]. The level of CPK required to confirm the diagnosis of rhabdomyolysis remains a matter of controversy, with suggested ranges above 5-10 times the upper limit of normal [2]. Etiologies of rhabdomyolysis are classified as either traumatic or non-traumatic with the latter encompassing drugs, toxins, infections, and metabolic, endocrine, and electrolyte disturbances, as well as inflammatory myopathies [3]. Recurrent episodes of rhabdomyolysis or very high levels of CPK (>50 times the upper limit of normal) may suggest the presence of metabolic myopathies [4,5]. We present a case of a young male patient with recurrent rhabdomyolysis with an extreme elevation of CPK.

## Case presentation

The patient was a 28-year-old man with a history of untreated bipolar disease who had initially presented to another hospital with fever, myalgia, dark urine, and respiratory symptoms of four days' duration. He had been diagnosed with pneumonia complicated by rhabdomyolysis and renal failure requiring hemodialysis. His CPK had reached nearly a million (900,000 IU/L) at the outside institution and only started to trend down after the dialysis. This was the patient’s third episode of rhabdomyolysis triggered by illness but the first one to require hemodialysis. He had experienced the first episode at 18 years of age.

The patient mentioned using over-the-counter medication for his symptoms: ibuprofen and a combination preparation of acetaminophen, dextromethorphan, phenylephrine, and doxylamine. He reported using the same over-the-counter medications prior to the last rhabdomyolysis episode he had experienced. He denied trauma or exertion, stated that he used marijuana only recreationally, and reported that he worked in plumbing. He had no family history of recurrent rhabdomyolysis or autoimmune disease. The laboratory findings are displayed in Table 1.

**Table 1:** Laboratory findings

Variables	Value/Result	Reference Range/Test
Creatinine Phosphokinase (CPK)	900,000 IU/L	<250 IU/L for Male
Human Immunodeficiency Virus (HIV)	Negative	Enzyme-Linked Immunosorbent Assay, Positive vs. Negative
Respiratory Viral Panel (BioFire)	Negative	BioFire Respiratory Panel PCR, Positive vs. Negative
Hepatitis Acute Panel (Hepatitis B Surface Antigen, Hepatitis B Surface Antibody, Hepatitis C Antibody)	Negative	Serological Assay, Positive vs. Negative
Legionella pneumophila Serogroup 1 Antigen	Negative	Enzyme Immunoassay, Positive vs. Negative
Cytomegalovirus PCR (Qualitative)	Negative	Real-Time Reverse Transcription-Polymerase Chain Reaction (RT-PCR), Positive vs. Negative
Urine Drug Screen	Positive for Cannabinoids	Positive vs. Negative
Antinuclear Antibody (ANA)	Negative	Enzyme-Linked Immunosorbent Assay, Positive vs. Negative
Antinuclear Aibonucleoprotein (Anti-U1 RNP)	Negative	Enzyme-Linked Immunosorbent Assay, Positive vs. Negative
Anti-Sjogren's Syndrome A (Anti-SSA), Anti-Sjogren's Syndrome B (Anti-SSB)	Negative	Enzyme-Linked Immunosorbent Assay, Positive vs. Negative
Smooth Muscle Antibodies (SMA)	Negative	Enzyme-Linked Immunosorbent Assay, Positive vs. Negative
Myositis Panel (PL-7, PL-2, Mi-2, Ku, Ej, Oj, Srp, Jo1) Autoantibodies	Negative	Enzyme-Linked Immunosorbent Assay, Positive vs. Negative
3-Hydroxy-3-Methylglutaryl-Coenzyme A Reductase Antibody (Anti-HMGCR)	Negative	Enzyme-Linked Immunosorbent Assay, Positive vs. Negative
Carnitine Ester/Free Ratio	0.7	0.1-0.8
Carnitine Esterified	66 umol/L	5-29 umol/L
Carnitine Free	94 umol/L	25-60 umol/L
Carnitine Total	160 umol/L	34-86 umol/L

The patient underwent a muscle biopsy that showed moderately active necrotizing myopathy. Features of inflammatory myopathy or vasculitis were not seen on the biopsy.

The patient was finally discharged with follow-up in the nephrology, rheumatology, and neurology outpatient clinics. Fortunately, the patient’s kidney function recovered, and dialysis was stopped. A rheumatology consultant concluded that there was no evidence of a rheumatologic condition. Neurology consultants plan to reassess a frozen muscle biopsy specimen to evaluate for metabolic myopathies.

## Discussion

Electrolyte disturbances, acute renal failure, and disseminated intravascular coagulation (DIC) are complications of rhabdomyolysis that warrant management in an intensive care unit [3]. Management with aggressive fluid hydration may cause respiratory compromise, and renal failure could require renal replacement therapy. In order to identify the cause of muscle injury, a thorough history is important to find out about any personal or family history of episodes, presence of trauma or exertion, and/or use of medications or illicit substances before symptom initiation.

This patient’s previous episodes of rhabdomyolysis had not been properly investigated, which warranted a workup for etiology. The patient did not use any high-risk medications that were known to cause rhabdomyolysis except for some over-the-counter medications. Doxylamine has been described in the literature to cause rhabdomyolysis when used excessively; however, our patient was compliant with the dosage limit [6-8]. Ibuprofen has been described in the literature to provoke rhabdomyolysis in patients with carnitine palmitoyltransferase II deficiency (CPT II) [9]. An elevated ratio of C16 + C18:1/C2 is characteristic of CPT II deficiency [10]. CPT II deficiency is considered a common cause of metabolic myopathies and is caused by mutations in the gene encoding for this enzyme involved in fatty acid oxidation [11,12]. Patients with the mild phenotype usually have symptoms in young adulthood and episodes of rhabdomyolysis are triggered by illness, emotional distress, exertion, and fever [13,14]. We speculate that our patient harbored this enzyme deficiency, but further genetic testing is needed to confirm this before the confirmation of a diagnosis.

In our review of the literature, only a few case reports were found to mention an extreme elevation of CPK to a level demonstrated by this patient [15-18]. Multiple studies have tried to show a correlation between the level of CPK and acute kidney injury (AKI) [19]. A recent meta-analysis by Safari et al. demonstrated a correlation between CPK and AKI in crush-induced rhabdomyolysis specifically [20]. Our patient required intermittent hemodialysis for one month before his renal function recovered.

## Conclusions

We described a case of severe rhabdomyolysis requiring renal replacement therapy and very high levels of CPK in a patient with a history of milder episodes of rhabdomyolysis. Recurrent cases of rhabdomyolysis and severe elevation in CPK raise concerns about an underlying metabolic myopathy as the culprit. Hence, recurrent rhabdomyolysis remains a diagnostic dilemma that requires careful investigation by healthcare providers to prevent future episodes and avert renal failure.
